# Prosecution for Alledged Mal-Practice

**Published:** 1848-10

**Authors:** 

**Affiliations:** Buffalo


ART. III.—*Prosecution for alledged Mal-Practice.*—Reported by Dr. HAMILTON.

*Supreme Court*—John C. Basset *vs.* John B. Collins and Anthony Barney.

In the fall of 1843, John C. Basset, of Independence, Allegany Co., aged 48, then in good health but corpulent, was injured by the upsetting of his wagon and the falling of a box, as was believed, upon his thigh. He was carried into a public house in Woodhull and there attended by Drs. Reed and Carey. After a careful and complete examination by measuring &c., they concluded that Mr. Basset had only received a severe bruise. He remained two weeks under their care and was then taken home in a bed. Four weeks after the accident Drs. Collins and Barney were called in, as the left leg was now said to be shortened and turned out. These gentlemen made an examination and found the leg in the following condition: shortened an inch and a half, the toes turned out, and could not be turned in, the left heel corresponding to the hollow of the right foot, a bunch in the groin like the head of the femur. They decided that it was a dislocation of the head of the femur upon the pubis, and with pulleys properly adjusted and carefully operated upon, proceeded to attempt its reduction. After two or three minutes' extension and counter-extension, a sound was heard, and a sensation felt by nearly all who were asssisting, which was was by them described as the sound and sensation usually produced when a dislocation is reduced. The patient was now released from the pulleys, and made to get up. The limb was of its original length and in its natural position, and the tumor in the groin had disappeared. The patient was again laid upon the bed and dismissed as cured.

It however appeared in testimony that a few days after, it was again shortened and turned out, but it does not appear that these facts came to the knowledge of the defendants: it also appeared that the plaintiff did not get the use of his limb so as to be able to dispense with crutches or a cane, in one or two years.

The limb is now shortened an inch and a half, and moderately turned out, but the motions of the joint are free and the plaintiff walks with a very slight halt and without inconvenience.

Drs. Collins and Barney were prosecuted and the case was tried in January, 1848, before Judge Marvin, but the jury having disagreed, it was again tried before Judge Mullett, in the circuit of the supreme court, held in August, 1848, in the town of Angelica, Allegany Co., N. Y.

In the first trial the plaintiff charged that the limb was sound when the defendants took hold of it with the pulleys, and that they then fractured it through the neck and without the capsule.

In the last trial this was not claimed, but it was alledged that the original accident was probably a fracture without the capsule and without displacement: that when examined by Drs. Collins and Barney a displacement had occurred and that the defendants were chargeable with criminal negligence or ignorance, in not discovering that it was a fracture; and consequently for subjecting the plaintiff to the useless pain of extension with the pulleys, and in not applying subsequently a retentive apparatus, since through this omission, the plaintiff has a shortened and crooked leg.

On the defence it was admitted that the original accident was a fracture without displacement, but that it was *within* the capsule and near the head of the bone; that its being within the capsule and near the head could alone satisfactorily account for the "bunch" in the groin, which disappeared with the reduction, and for the slowness of the subsequent restoration of the limb. It was claimed, also, that the signs described by the witnesses were the ordinary signs of a dislocation upon the pubis, and would be likely to deceive the most skillful surgeon; that several eminent surgeons had mistaken fractures of the thigh for dislocations; that the extension with the pulleys did him no permanent injury; that the subsequent treatment pursued by the patient in this case, viz: keeping his bed for a few days and then getting about on crutches, would have been the proper treatment had the exact nature of the accident been fully known, and finally, that the patient has as good a limb as can ordinarily be expected in this fracture under the most skillful management.

The examination of the numerous witnesses having closed, the jury were addressed by the distinguished gentlemen, Messrs. Doolittle and Skinner for the plaintiff, and Messrs. Grover and Peck for the defendant; and the Hon. Judge followed with a most pungent and impressive charge, in which the jury were instructed to disregard all mere appeals to their prejudices, and especially to reject that counsel which would advise them to look upon the medical profession as an *oppressive and aristocratic monopoly*, and to decide the case upon the facts as drawn from the witnesses upon the stand.

The jury retired and in a few minutes returned a verdict for the defendants.

The defendants in this case, were men who have long practised both medicine and surgery in the county of Allegany. Dr Barney at Independence, and Dr Collins at Alfred; and they both occupy a high position in the estimation of the public, as men of skill and worth. Dr. Collins was for some years an active member of the state legislature; and it is gratifying to know that in the mind of the Hon. Judge, as well as of the very intelligent jury, they received a full and unqualified acquittal from the charge of any degree of negligence or unskilfulness.

The medical witnesses examined on the part of the plaintiff, were Drs. John Read, of Woodhull, John R. Hartshorn, of Alfred, John H. Thorp, of Independence, and Charles D. Robinson, of Almond, all of Allegany Co., and Dr. Abijah Case, of Howard, Steuben Co. On the part of the defence were Drs. Erastus Willard, of Friendship, Richard Charles, of Angelica, Randall Reed, of Amity, George B. Jones, of Scio, Lorenzo Dana, of Friendship, and Walter Wallace, of Angelica, all of Allegany Co., and Dr. Frank H. Hamilton, of Buffalo, Erie Co.

There are several instances on record, where distinguished surgeons have mistaken a fracture at the neck of the femur for dislocation, and we have no doubt there have occurred thousands of similar errors in diagnosis, which have not been recorded, and which need only the friendly advice of some humane doctor, whispered in the ear of the unfortunate victim of the mistake, to bring them to light.

Sabatier, a distinguished Parisian surgeon, confesses that he had a case of fracture of the neck of the femur, without displacement, but in which he declared there was neither fracture nor dislocation. Some days after, shortening occurred, and he discovered his mistake. Ambrose Pare, surgeon to Henry II of France, and to three succeeding French kings, the most distinguished surgeon of his age, says that he attended a lady whose thigh he thought was dislocated, and accordingly he attempted to reduce it, and actually believed he had done so. Two days after, the limb was again shortened and the foot turned in, as at first; he again attempted to reduce it, but having accidentally discovered crepitus, the error was apparent. John Petit, director of the Academy of Surgery in Paris, and a great surgeon in his day, says that he diagnosed a fracture of the neck of the femur as a dislocation upward and backward, but at length discovered his error. Mr. Stanley, of London, relates a case in which a lad of 18 years fell, and was supposed to have dislocated the head of the femur into the foramen ovale, for the thigh was bent at a right angle with the body, and could by no means be extended; abduction was difficult; the toes were everted; the limb appeared to be lengthened; there was no crepitus.—From all these circumstances, together with the age of the patient, it was naturally enough concluded that it was a dislocation. Accordingly, the pulleys were applied and extension made. Three months after he died of small pox in St. Bartholomew's hospital, London, and the post-mortem revealed the following facts: "the capsule was entire, but a little thickened; the ligamentum teres was uninjured, (of course no dislocation had ever occurred,) a line of fracture extended obliquely through the neck of the femur, and entirely within the capsule; the neck was shortened, but the fractured surfaces were in the closest apposition, and united nearly in their whole extent by bone, with an irregular callous around the seat of the fracture."

BUFFALO, Sept. 1848.

